# Metabolic reprogramming and immune evasion interaction in the tumor microenvironment promote tumor progression

**DOI:** 10.3389/fonc.2026.1896937

**Published:** 2026-08-28

**Authors:** Hailun Wang, Hanyu Zhao, Xuelu Pu, Yandu He, Yajun Li

**Affiliations:** 1 Department of Oncology, The Third Affiliated Hospital of Zunyi Medical University (The First People’s Hospital of Zunyi), Zunyi, China; 2 Department of Oncology, Zunyi Medical University, Zunyi, China; 3 Department of Oncology, The Eighth Affiliated Hospital, Southern Medical University (The First People’s Hospital of Shunde Foshan), Foshan, China

**Keywords:** clinical translation, combined therapy, immune evasion, immunosuppressive microenvironment, metabolic reprogramming, metabolism-immunity axis, tumor microenvironment, tumor progression

## Abstract

Emerging evidence demonstrates that tumor metabolic reprogramming not only supports tumor-cell proliferation but also promotes the establishment of an immunosuppressive tumor microenvironment (TME) by altering nutrient competition and metabolite accumulation. Therefore, metabolic reprogramming and immune evasion should be regarded as closely interconnected processes rather than independent phenotypes. By altering the metabolite composition of the TME and local metabolic programs, tumor metabolic reprogramming suppresses immune activation and shapes immune-cell function and fate, thereby promoting cancer immune evasion. This review focuses on two mechanistically developed axes linking tumor metabolism to immune suppression: glycolysis-associated lactate accumulation and lactylation, and nutrient competition involving amino acids and lipids. We summarize how lactate acts as both a metabolic substrate and signaling mediator, how lactylation translates metabolic changes into epigenetic regulation of immune-related transcriptional programs, and how depletion of glutamine, tryptophan, and arginine, together with lipid accumulation and remodeling, impairs effector-cell metabolic fitness while favoring regulatory T cells, tumor-associated macrophages, and myeloid-derived suppressor cells. We further examine hypoxia as a contextual amplifier, the immune-evasion outcomes and reciprocal feedback circuits produced by these alterations, and therapeutic strategies targeting the metabolism–immunity axis. Particular attention is given to context-dependent effects and evidence maturity, because lactate-, hypoxia-, and metabolite-associated pathways are not uniformly immunosuppressive across cell types and conditions. Although preclinical findings support interventions targeting lactate production or transport, lactylation-associated regulators, amino acid metabolism, and the ecto-5′-nucleotidase (CD73)–adenosine axis, clinical evidence remains limited and heterogeneous. Biomarker-guided patient selection, confirmation of target engagement, preservation of immune-cell metabolic fitness, and rational combination strategies will be essential for clinical translation.

## 1 Introduction

Metabolic reprogramming and immune evasion are critical processes underlying tumor progression and therapeutic resistance. Rather than functioning as independent events, metabolic alterations within tumors continuously interact with immune regulation in the tumor microenvironment (TME). To support proliferation, survival, and adaptation to environmental stress, tumor cells undergo extensive metabolic remodeling, which alters nutrient availability and metabolite composition within the TME and subsequently affects antitumor immune responses (1–3). Increasing evidence indicates that metabolic competition and metabolite accumulation can impair the function of immune effector cells while supporting immunosuppressive cell populations, thereby contributing to immune escape (4, 5). Thus, metabolic reprogramming and immune evasion represent interconnected processes that jointly shape the immunological landscape of tumors.

Among the diverse metabolic alterations observed in tumors, enhanced glycolysis, lactate accumulation, and nutrient competition have emerged as important mechanisms linking tumor metabolism with immune suppression. Lactate, traditionally considered a by-product of glycolysis, is now recognized as an active metabolic signal that can regulate cellular functions within the TME (5, 6). The discovery of lactylation further revealed that lactate metabolism can be directly connected to epigenetic regulation, providing a mechanism through which metabolic changes influence gene expression programs associated with tumor progression and immune regulation (7, 8). In parallel, competition for amino acids, including glutamine, tryptophan, and arginine, between tumor cells and immune cells can restrict nutrient availability, alter immune-cell metabolic states, and impair antitumor activity (4, 9, 10). These findings highlight lactate/lactylation and nutrient availability as important components of metabolic regulation underlying tumor immune evasion.

**Figure 1:**
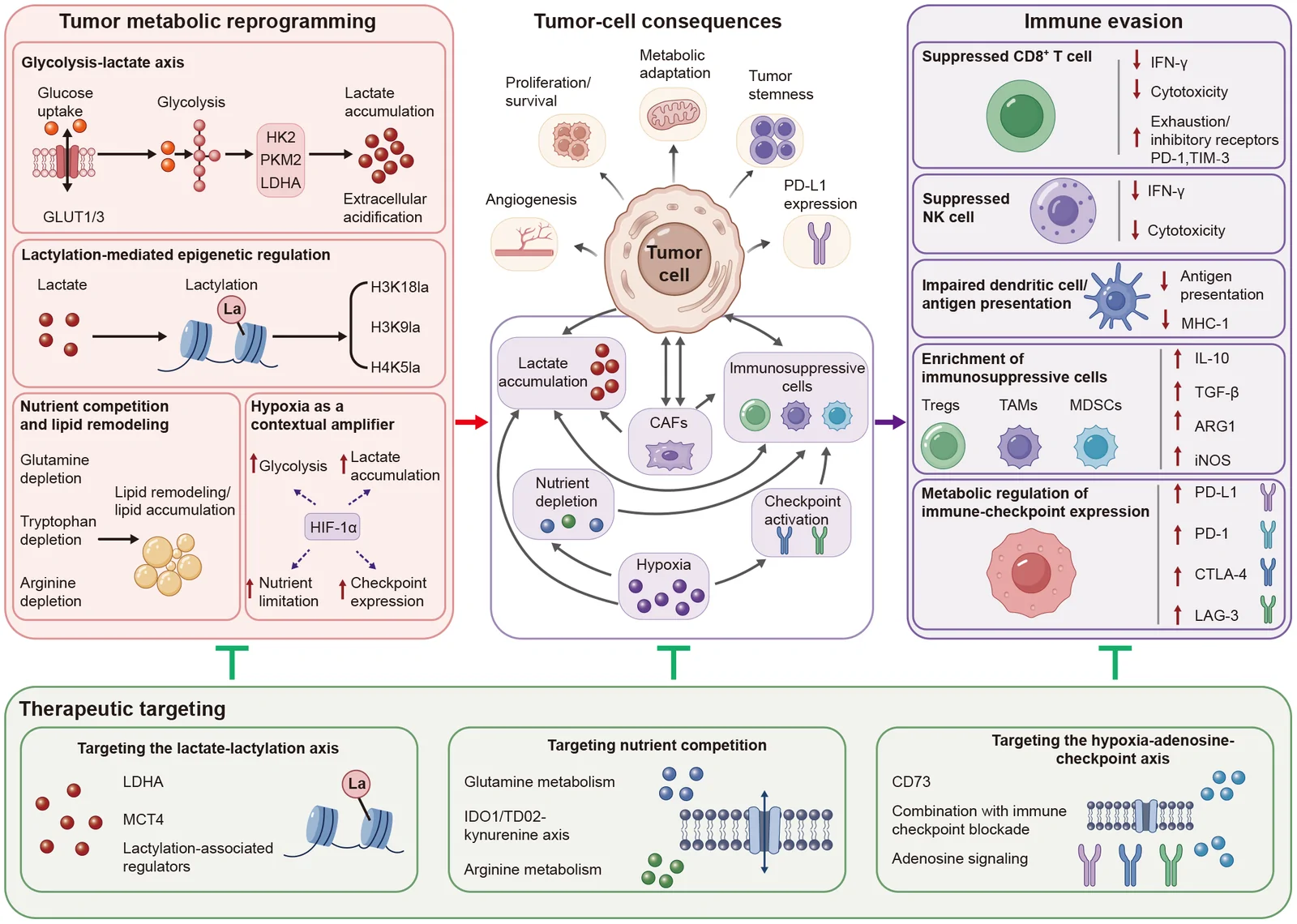
Overview of tumor metabolic reprogramming and immune evasion in the tumor microenvironment. The figure summarizes the major metabolic alterations, downstream consequences, immune-evasion mechanisms, and therapeutic strategies involved in tumor metabolic reprogramming. The left panel illustrates key metabolic changes, including the glycolysis–lactate axis, lactylation-mediated epigenetic regulation, nutrient competition, lipid remodeling, and hypoxia-associated adaptation. The central panel depicts how these metabolic alterations reshape the tumor microenvironment and promote tumor-cell survival, stemness, angiogenesis, immune checkpoint activation, and expansion of immunosuppressive populations. The right panel summarizes major immune-evasion outcomes, including impaired CD8^+^ T-cell, NK-cell, and dendritic-cell function, enrichment of immunosuppressive cells, and metabolic regulation of immune checkpoints. The bottom panel presents potential therapeutic strategies targeting the lactate/lactylation axis, nutrient competition, and hypoxia–adenosine–checkpoint signaling pathways. HK2, hexokinase 2; PKM2, pyruvate kinase M2; LDHA, lactate dehydrogenase A; HIF-1α, hypoxia-inducible factor 1α; CAFs, cancer-associated fibroblasts; Tregs, regulatory T cells; TAMs, tumor-associated macrophages; MDSCs, myeloid-derived suppressor cells; PD-L1, programmed death-ligand 1; MHC-1, major histocompatibility complex class I; ARG1, arginase 1; iNOS, inducible nitric oxide synthase; CD73, ecto-5′-nucleotidase; CTLA-4, cytotoxic T-lymphocyte-associated protein 4; LAG-3, lymphocyte activation gene-3.

Previous reviews have examined cancer immune evasion through broad conceptual frameworks, summarized the metabolic characteristics and therapeutic vulnerabilities of tumors, or discussed how metabolic reprogramming in tumor and immune cells affects antitumor immunity and immunotherapy (11–13). More recent reviews have further provided comprehensive accounts of immune-evasion mechanisms or described how altered glucose, amino acid, and lipid metabolism in tumor and non-tumor cells contributes to an immunosuppressive TME (14, 15). Building on this literature, the present review does not seek to establish metabolism–immune evasion as a previously unrecognized connection. Instead, it places particular emphasis on lactate/lactylation and nutrient competition as two mechanistically developed axes and organizes the evidence from core metabolic alterations, through immune-evasion outcomes and reciprocal feedback circuits, to therapeutic intervention and barriers to clinical translation. We first summarize glycolysis-associated lactate accumulation and signaling, lactylation-mediated epigenetic regulation, and competition for amino acids and lipids. We then examine how these alterations impair effector-cell function, antigen presentation, and immune-checkpoint regulation, reinforce immunosuppressive-cell adaptation, and form self-sustaining metabolic–immune circuits. Finally, we evaluate therapeutic strategies targeting these circuits, with attention to evidence maturity and translational limitations (**Figure 1**).

## 2 Core patterns and molecular mechanisms of tumor metabolic reprogramming

### 2.1 Glycolysis, lactate accumulation, and lactate signaling

The Warburg effect describes a metabolic phenotype in which tumor cells preferentially depend on glycolysis rather than oxidative phosphorylation (OXPHOS) for energy production, even under relatively oxygen-sufficient conditions (16). Through enhanced glucose uptake, glycolytic flux, and lactate production, tumor cells generate the energy and biosynthetic intermediates necessary for rapid proliferation while maintaining metabolic adaptation to hypoxic or nutrient-deprived environments (17). Beyond supporting energy metabolism, the Warburg effect also alters local acid–base balance and metabolite composition, thereby reshaping the functional state of TME (18). First described by Otto Warburg, this phenomenon is now recognized as one of the hallmark features of tumor metabolic reprogramming (19). Increasing evidence further suggests that the Warburg effect is not merely an intracellular metabolic alteration but is also closely associated with tumor growth, invasion, metastasis, and the establishment of an immunosuppressive microenvironment (14). Consequently, the Warburg effect provides a central framework for understanding tumor metabolic dysregulation and serves as an important foundation for investigating the interplay among glucose metabolism, lactate signaling, and immune regulation.

The establishment and maintenance of the Warburg phenotype rely largely on the coordinated regulation of multiple glycolytic enzymes. Hexokinase 2 (HK2) catalyzes the first phosphorylation step of glucose metabolism, thereby retaining glucose within the cell and directing it into the glycolytic pathway (19). Pyruvate kinase M2 (PKM2) regulates glycolytic flux through distinct conformational transitions and controls the redistribution of intermediate metabolites into anabolic pathways required for tumor growth (20). Lactate dehydrogenase A (LDHA) catalyzes the conversion of pyruvate into lactate, thereby sustaining elevated glycolytic activity (18). In addition to conventional enzymatic regulation, aerobic glycolysis is also influenced by epigenetic and transcriptional regulatory mechanisms, which contribute substantially to the metabolic heterogeneity observed across different tumor types (21, 22).

Lactate is one of the most important metabolites generated during the Warburg effect; however, it is no longer viewed merely as a terminal product of glycolysis. Instead, lactate is now recognized as a critical mediator linking the functional states of tumor cells and immune cells within TME (5, 6). Under physiological conditions, lactate can be reconverted into pyruvate and further incorporated into mitochondrial metabolism; however, within TME, lactate is frequently exported through monocarboxylate transporter 4 (MCT4) and subsequently taken up by neighboring cells via monocarboxylate transporter 1 (MCT1), thereby establishing a form of transcellular metabolic coupling (23). Beyond serving as a metabolic substrate, lactate also functions as an important signaling molecule, with its biological effects varying according to cell type, oxygen availability, and microenvironmental conditions (24). Mechanistically, lactate can inhibit pyruvate carboxylase activity, redirect pyruvate metabolic flux, induce metabolic reprogramming in T cells, interfere with T cell receptor (TCR) signaling, and disrupt intracellular NAD+/NADH balance, ultimately impairing CD8^+^ T cell proliferation, cytotoxic activity, and tumor-recognition capacity (25–27). In parallel, acidification of TME further promotes tumor-associated macrophage (TAM) polarization toward an M2-like phenotype, reduces NKp46 expression, and suppresses interferon-γ (IFN-γ) secretion and T cell activation, thereby aggravating local immunosuppression (28). Moreover, lactate released by tumor cells in hypoxic regions can be reused by cells located in relatively oxygenated areas, thereby supporting metabolic cooperation, sustaining energy supply, and promoting angiogenesis and immune evasion (29).

Lactate can further facilitate tumor proliferation, migration, and immune regulation by altering acid–base homeostasis, stabilizing hypoxia-inducible factor 1α (HIF-1α), and activating signaling pathways such as GPR81 (30, 31). These processes are tightly regulated by transcription factors such as HIF-1α and c-Myc and occur concurrently with enhanced glucose uptake and increased expression of HK2 and LDHA (32, 33). Increasing evidence indicates that lactate functions both as a mediator of metabolic coupling and a central signaling node connecting metabolic reprogramming with immunosuppression (34). Within highly glycolytic TME, lactate directly impairs the effector functions of CD8^+^ T cells while simultaneously supporting the survival and activity of immunosuppressive cell populations, including regulatory T cells (Tregs), myeloid-derived suppressor cells (MDSCs), and TAMs (28).

### 2.2 Lactylation and epigenetic regulation

Lactylation, a lactate-mediated post-translational modification involving both histone and non-histone proteins, has recently attracted considerable attention (23). Mechanistically, lactate-derived substrates generated through LDH- and monocarboxylate transporter (MCT)-associated metabolic pathways can enter the nucleus and undergo catalysis by lactyltransferases such as p300/CBP, KAT8, and AARS1. This process alters lysine-residue conformation, chromatin accessibility, and protein function, thereby contributing to tumor stemness and immune evasion. Conversely, histone deacetylases (HDACs) and sirtuins can mediate delactylation, reversing these modifications and thereby suppressing aberrant transcriptional programs and oncogene expression (7, 8). This mechanism directly links changes in metabolic state to transcriptional remodeling, thereby coupling metabolic reprogramming with epigenetic regulation. Lactylation at sites such as H3K18la, H3K9la, H3K56la, and H4K12la can influence chromatin accessibility and, together with oncogenic pathways such as PI3K/AKT and MAPK signaling, regulate genes involved in tumor proliferation, metastasis, and immune evasion (35, 36). Ma Z et al. further reported that lactate promotes B7-H3 expression through enhanced H3K18 lactylation, thereby reducing the infiltration and functional activity of CD8^+^ T cells within tumors (37).

Although lactate itself does not directly bind gene promoters, lactate-mediated histone lactylation marks, including H3K18la and H3K9la, have been identified in gliomas and are associated with altered expression of immunosuppression-related genes such as retinoic acid receptor γ, iNOS, and IL-12 (38, 39). Lactate-mediated H3K18la and H3K9la can promote TAM polarization toward an M2-like phenotype while reducing expression of IL-12p40, the costimulatory molecule CD80, and downstream targets of X-box binding protein 1, thereby inhibiting dendritic cell (DC) activity (39). Increased lactate production mediated by LDHA promotes E1A binding protein p300 (EP300)-mediated H4K5la formation and directly activates programmed death-ligand 1 (PD-L1) transcription, thereby constituting the LDHA–lactate–EP300–H4K5la–PD-L1 regulatory axis (40). In parallel, Warburg effect-associated acidification of TME not only inhibits the activity of CTLs, DCs, and natural killer (NK) cells but also suppresses effector cytokines such as IFN-γ and promotes macrophage polarization from the proinflammatory M1 phenotype toward the anti-inflammatory M2 phenotype through regulation of epigenetic modifiers such as HDACs, EZH2, and JMJD (38).

### 2.3 Nutrient competition and availability involving amino acids and lipids

Nutrient deprivation and metabolic competition represent fundamental mechanisms underlying the establishment of an immunosuppressive TME. These processes are characterized by the ability of tumor cells to exploit their metabolic advantages, preferentially consume local substrates, and actively reshape the immune microenvironment. In contrast, immunosuppressive cells such as Tregs, TAMs, and MDSCs possess greater metabolic flexibility and can maintain survival and suppressive activity through pathways such as fatty acid oxidation (FAO), the Warburg effect, or amino acid metabolic remodeling (41, 42).

#### 2.3.1 Amino acid competition and depletion

Abnormal glutamine metabolism represents one of the most prominent features of amino acid metabolic reprogramming in tumors (10). As a major source of carbon and nitrogen, glutamine supports tumor-cell biosynthesis and energy metabolism through its involvement in the tricarboxylic acid (TCA) cycle, nucleotide synthesis, and redox homeostasis (43). To sustain rapid proliferation, tumor cells commonly increase glutamine uptake and catabolism, thereby maintaining a highly active metabolic state. The c-MYC-driven “glutamine addiction” phenotype enhances glutamine utilization in tumor cells through upregulation of alanine-serine-cysteine transporter 2 and GLS, thereby continuously suppressing T cell activity (4).

Aberrant tryptophan metabolism represents a classical mechanism through which tumors establish immunosuppression via amino acid metabolic reprogramming (44). Indoleamine 2,3-dioxygenase 1 (IDO1) and tryptophan 2,3-dioxygenase (TDO2) promote tryptophan degradation through the kynurenine (Kyn) pathway, resulting in tryptophan depletion and Kyn accumulation within TME, thereby suppressing T cell activity and enhancing immune tolerance (9). The immunosuppressive effects of tryptophan depletion and Kyn accumulation involve both nutrient-sensing and receptor-mediated signaling pathways. On the one hand, tryptophan depletion induces T cell cycle arrest and functional exhaustion through activation of general control nonderepressible 2 and inhibition of mechanistic target of rapamycin complex 1 (mTORC1). On the other hand, Kyn accumulation promotes Treg differentiation and MDSC recruitment through activation of the Kyn/aryl hydrocarbon receptor (AhR) signaling axis (45, 46). Furthermore, downstream Kyn metabolites, including 3-hydroxyanthranilic acid and quinolinic acid, can directly induce CD8^+^ T cell apoptosis and suppress CD8^+^ T cell activation (47, 48).

Tumor cells can induce high expression of arginase 1/2 (ARG1/2) in MDSCs and M2-TAMs, thereby promoting the conversion of arginine into ornithine and urea. These metabolites subsequently support polyamine and proline synthesis, contributing to cell proliferation and collagen production (49). Simultaneously, ARG1-mediated arginine depletion and enhanced iNOS-dependent nitrogen metabolism can generate excessive reactive oxygen species (ROS)/reactive nitrogen species and induce downregulation of the TCR CD3ζ chain. Arginine deprivation can also suppress mTOR signaling, leaving CD8^+^ T cells in a metabolically deficient state and ultimately impairing their proliferation and cytotoxic activity (50, 51).

#### 2.3.2 Lipid availability, accumulation, and immune-cell adaptation

Under conditions such as hypoxia, nutrient deprivation, and oxidative stress, tumor cells undergo extensive lipid metabolic remodeling as an adaptive strategy to sustain survival and proliferation (52). This remodeling is primarily characterized by enhanced *de novo* lipid synthesis, increased uptake of exogenous lipids, and activation of fatty acid oxidation (FAO), thereby meeting the demands of membrane biosynthesis, signaling-molecule production, ATP generation, and NADPH maintenance (53–55).

Specific lipid metabolites can directly alter immune-cell function and indirectly promote myeloid immunosuppressive phenotypes, thereby collectively facilitating the formation of an immunosuppressive microenvironment (28). Oncogenic KRAS signaling promotes metabolic reprogramming and cholesterol accumulation in the tumor microenvironment, which contributes to CD8^+^ T cell exhaustion and functional suppression through mechanisms involving ER stress-induced XBP1 activation and oxysterol-mediated LXR signaling (50). Through the UDP-glucose ceramide glucosyltransferase-β-1,4-galactosyltransferase. 5-lactosylceramide axis, glycosphingolipid (GSL) metabolism contributes to the maintenance of normal immune synapse integrity and survival of NK cells and CD8^+^ T cells and may also promote immune evasion when aberrantly remodeled (56). Dual-specificity phosphatase 18 in effector T cells can promote lanosterol accumulation and extracellular release through the upstream stimulatory factor 1–SREBF2 signaling axis (57). After uptake by CD8^+^ T cells, lanosterol suppresses the mevalonate pathway and inhibits KRAS/ERK-related protein prenylation, ultimately impairing T cell activation (58).

Lipid metabolic remodeling further reinforces the metabolic advantages of immunosuppressive cells. Under nutrient-restricted conditions such as hypoxia and glucose deprivation, TAMs can extensively uptake cholesterol, fatty acids, and phospholipids derived from tumor-associated metabolic dysregulation, thereby promoting polarization toward a tumor-supportive M2-like phenotype (59, 60). Tregs can also adapt to substrate-limited conditions by enhancing FAO, thereby stably maintaining their immunosuppressive functions (61). MDSCs often exhibit simultaneous enhancement of glycolysis and FAO and can potently suppress CD8^+^ T cell responses (41).

Collectively, tumor-cell nutrient use and lipid remodeling reduce the metabolic support available to effector immune cells while favoring the adaptation of immunosuppressive cell populations. Amino acid depletion and lipid or metabolite accumulation therefore impairs antitumor T and NK cell functions, whereas Tregs, TAMs, and MDSCs maintain immunosuppressive phenotypes, collectively facilitating *immune evasion.*

### 2.4 Hypoxia as a contextual amplifier

Rather than constituting a separate mechanistic axis, hypoxia acts as a contextual amplifier by intensifying glycolysis and lactate accumulation, nutrient limitation, and immune-checkpoint expression. HIF-1α is a central transcriptional regulator that enables tumor cells to adapt to hypoxic microenvironments (62). Under hypoxic conditions, HIF-1α promotes the metabolic transition from OXPHOS to glycolysis and enhances tumor-cell survival by upregulating genes involved in glucose uptake, lactate production, antioxidant defense, and angiogenesis (63, 64). At the molecular level, HIF-1α induces the upregulation of glucose transporter 1 (GLUT1)/glucose transporter 3 (GLUT3) and HK2, thereby enhancing glucose uptake and glycolytic activity (65). HIF-1α also upregulates LDHA and MCT4, thereby increasing lactate production and export, promoting extracellular acidification, and reinforcing adaptation under hypoxic stress (66–70). Thus, hypoxia amplifies the glycolytic and lactate-rich metabolic state described above.

These hypoxia-driven metabolic alterations reshape the acidic and nutrient-competitive landscape of TME and exert unequal effects on different immune-cell populations (71, 72). Mechanistically, coordinated regulation by HIFs, mTOR, the unfolded protein response, and nuclear factor-κB (NF-κB) enables tumor cells to maintain metabolic plasticity under conditions of lactate accumulation, oxidative stress, and nutrient deprivation (73–75). These adaptations not only reshape the acidic and nutrient-competitive landscape of TME but also impair antitumor immunity by disrupting the metabolic fitness and functional adaptation of immune cells (76). CD36, which is highly expressed in both human and mouse tumor tissues, enhances lipid uptake by Tregs within TME and activates peroxisome proliferator-activated receptor-β signaling. This process increases mitochondrial OXPHOS, elevates the NAD/NADH ratio, and maintains expression of molecules such as glucocorticoid-induced TNFR-related protein and OX40, thereby supporting Treg survival and immunosuppressive activity (59). Consistently, Treg-specific CD36-knockout mice exhibit significantly increased Treg apoptosis (59). Another study using multiple tumor-bearing mouse models further demonstrated that hypoxia suppresses the core lipid-synthesis transcription factors SREBP1/2 and reduces expression of acetyl-CoA carboxylase alpha, FASN, and HMGCR, ultimately impairing lipid synthesis in CD8^+^ T cells (77). Collectively, hypoxia-induced metabolic stress favors the adaptation of immunosuppressive cells while compromising effector-cell function.

In addition to these indirect effects mediated by metabolic stress, hypoxia can directly activate immune-checkpoint-associated programs. Hypoxia-induced immunosuppression reflects the combined effects of metabolic dysregulation, signaling-network remodeling, and altered intercellular communication (78). Existing evidence consistently demonstrates an association between hypoxia and PD-L1 upregulation, with signaling pathways such as HIF-1α, STAT3, and NF-κB playing central roles, although the specific molecular mechanisms vary across tumor models (79). Hypoxia can upregulate PD-L1 through HIF-1α-dependent and related pathways, thereby inhibiting T cell activation and cytotoxicity. Hypoxia can also interact with other immune-checkpoint molecules, such as cytotoxic T-lymphocyte-associated protein 4 (CTLA-4) and lymphocyte-activation gene 3 (LAG-3), further strengthening the immunosuppressive network (80, 81). Li L et al. analyzed 75 lung adenocarcinoma samples and showed that hypoxia-induced glycogen branching enzyme 1 is highly expressed in tumor tissues. Glycogen branching enzyme 1 upregulation can inhibit fructose-1,6-bisphosphatase 1 and stabilize HIF-1α expression, thereby establishing a positive-feedback loop that ultimately promotes immune escape (82). In a pancreatic cancer xenograft mouse model, hypoxia increased PD-L1 expression by activating the GLS2/YAP1 signaling axis while simultaneously suppressing CD4^+^ and CD8^+^ T cell activation (83).

Importantly, the immunoregulatory role of HIF-1α exhibits substantial context dependency. On the one hand, HIF-1α can upregulate molecules such as PD-L1, V-domain Ig suppressor of T cell activation, CCL28, and miR-224, act on multiple immune-cell types, including T cells, NK cells, and macrophages, and thereby promote immune escape under hypoxic conditions (84). On the other hand, HIF-1α-mediated metabolic adaptation may facilitate immune-cell survival and functional maintenance in low-oxygen environments (85). For example, in lung tissues, HIF-1α helps maintain the survival of CD4^+^ tissue-resident T cells, promotes IL-21 secretion, and upregulates CXCR6 expression, suggesting an important role in local immune homeostasis (86). Therefore, the biological effects of hypoxia depend on cell type and microenvironmental context. Overall, hypoxia links and amplifies glycolysis and lactate accumulation, nutrient limitation, immune-cell metabolic adaptation, and checkpoint activation rather than constituting an independent mechanistic framework.

## 3 Key immune-evasion outcomes associated with tumor metabolic reprogramming

### 3.1 Effector cell dysfunction

Inhibition of immune effector-cell function represents one of the most direct manifestations of tumor immune evasion and is primarily characterized by the progressive dysfunction and eventual exhaustion of antitumor effector cells, particularly CTLs (87). Under conditions of persistent antigen exposure and continuous inhibitory signaling, tumor-infiltrating T cells commonly exhibit reduced secretion of effector cytokines, impaired cytotoxic activity, and sustained upregulation of inhibitory receptors such as programmed cell death protein 1 (PD-1) and T cell immunoglobulin and mucin domain-containing protein 3 (TIM-3) (88). This dysfunction is not limited to classical exhaustion but may additionally involve broader impairments, such as spatial exclusion and compromised immune surveillance. In epidermal growth factor receptor (EGFR)-mutant non–small-cell lung cancer (NSCLC), the HHLA2–KIR3DL3 signaling axis promotes T cell exhaustion through reprogramming of amino acid metabolism (89). Similarly, in colorectal cancer (CRC), downregulation of hepatocyte nuclear factor 4α and its downstream target ornithine transcarbamylase disrupts the urea cycle and leads to ammonia accumulation. This metabolic alteration suppresses the trans-sulfuration pathway in T cells, reduces glutathione (GSH) synthesis, induces oxidative stress, upregulates exhaustion-associated molecules such as PD-1 and CTLA-4, and ultimately inhibits proliferation of both CD4^+^ and CD8^+^ T cells (90).

Metabolic dysregulation and redox imbalance together constitute a major molecular basis underlying immune effector-cell dysfunction. Methylmalonic acid, a metabolite generated during propionate oxidation, can upregulate the exhaustion-associated regulator thymocyte selection-associated high mobility group box protein and induce mitochondrial dysfunction, thereby weakening antitumor immune responses (91). Adenosine suppresses IFN-γ, TNF-α, and GZMB secretion by CD8^+^ T cells and impairs their cytotoxic activity (92). In NK cells, adenosine interferes with IL-12/IL-15-related signaling, suppresses OXPHOS and glycolysis, induces metabolic remodeling, and weakens antitumor cytotoxicity (15). Similarly, lactate accumulation and extracellular acidification can reduce NKp46 expression and suppress NK cell activation and cytotoxic function, further compromising antitumor immune surveillance (28, 93). Together, these findings suggest that impairment of immune effector-cell function is a progressive and dynamic process driven by the combined effects of inhibitory receptor signaling, metabolic imbalance, redox imbalance, and TME remodeling.

### 3.2 Impaired dendritic cell function and antigen presentation

Impaired antigen presentation represents a fundamental mechanism underlying tumor immune escape. Rather than resulting from a single molecular defect, this process reflects the combined effects of major histocompatibility complex class I (MHC-I) downregulation, dysfunction of antigen-processing pathways, and establishment of a metabolically suppressive microenvironment, ultimately leading to reduced tumor-antigen visibility, weakened T cell recognition, and diminished responsiveness to ICIs (14, 94). MHC-I loss or downregulation occurs in various tumors, although its frequency varies according to cancer type, detection methodology, and evaluation criteria (95).

DCs, which serve as the most important professional antigen-presenting cells, are highly sensitive to metabolic alterations within TME (92, 96). Impaired lactate catabolism and abnormal accumulation or depletion of metabolites such as lipids and amino acids can directly disrupt antigen processing and presentation, as evidenced by reduced activity of antigen-processing enzymes and transporter associated with antigen processing (97). A high-lactate microenvironment impairs differentiation of monocytes into mature DCs and suppresses DC activation and migration, thereby reducing the efficiency of tumor-antigen presentation to T cells (18, 30). Oxysterols can regulate macrophage lipid phagocytosis through LXR signaling and impair MHC class II-mediated antigen presentation to helper T cells (98).

Metabolic stress can also affect antigen presentation through more specific molecular pathways. In kidney renal clear cell carcinoma, Parkin deficiency induces mitochondrial metabolic stress, leading to phosphatase and tensin homolog degradation and overactivation of the PTEN/Akt-signaling pathway, thereby suppressing the MHC-I antigen-presentation pathway (99). mGluR4 induces metabolic reprogramming and functional impairment in DCs by inhibiting AC/PKA, NF-κB, and PI3K–Akt-signaling pathways; in mGluR4-deficient mouse models, DC migration, maturation, antigen processing, and antigen presentation were enhanced (100). Zeb1 regulates the cross-presentation capacity of type 1 conventional dendritic cells through the microRNA-96/182–Cybb pathway, whereas Zeb1 deficiency results in reduced cDC1 abundance and impaired function (101).

Lipid metabolic remodeling further aggravates antigen-presentation defects. Fatty acid-binding protein 5 in Tregs promotes nuclear lipid transport and enhances lipid metabolism in plasmacytoid DCs, thereby driving polarization toward a tolerogenic phenotype and weakening DC antigen-presentation capacity (102). Abnormal accumulation of triglycerides and low-density lipoprotein interferes with transport of antigen peptide–MHC-I complexes to the cell membrane, promotes tolerogenic DC formation, enhances IL-10 secretion and Treg activation, and ultimately impairs DC maturation and CD8^+^ T cell activation (103). Reducing lipid accumulation in tumor-infiltrating DCs increased the proportion of mature intratumoral DCs, promoted CD8^+^ T cell infiltration, and enhanced cytokine secretion trends in mouse models of colon cancer and melanoma (104).

Metabolite-mediated regulation of antigen presentation is not always uniformly suppressive but depends on metabolite concentration and microenvironmental context (105). At low concentrations, succinate can cooperate with Toll-like receptor signaling to promote DC maturation and migration; in contrast, excessive succinate accumulation can limit DC over maturation through HIF-1α, reduce proinflammatory cytokine release, and shift DCs toward an immune-tolerant phenotype (106, 107). These findings indicate that antigen-presentation defects result from mutual reinforcement between metabolic imbalance and reduced antigen visibility.

### 3.3 Enrichment of immunosuppressive cells

Another defining feature of TME is the accumulation of immunosuppressive cell populations, which reflects the transition from localized molecular abnormalities to global remodeling of the immune ecosystem. The increased abundance of Tregs, TAMs, and MDSCs within tumor tissues involves not only expansion in cell number but also coordinated reorganization of spatial distribution, metabolic activity, and intercellular communication networks (108). These cells collectively restrict CD8^+^ T cell infiltration and activation through secretion of inhibitory cytokines, competition for essential nutrients, and remodeling of the extracellular matrix (109).

Tregs and M2-TAMs secrete immunosuppressive cytokines such as IL-10 and TGF-β, whereas CAF-associated extracellular-matrix remodeling and CXCL12 signaling restrict T cell migration and favor the accumulation of immunosuppressive populations (21, 22, 93). TAM-derived 25-hydroxycholesterol can further promote macrophage polarization toward an immunosuppressive phenotype through the GPR155–mTORC1–AMPKα–STAT6-signaling axis (110).

Spatial heterogeneity further enhances the stability of this immunosuppressive ecosystem. MDSCs and Tregs preferentially accumulate at the tumor invasive margin and within hypoxic regions, and this distribution pattern is associated with increased expression of PD-L1 and CTLA-4 (41). In CRC, folate receptor beta 2-positive and lymphatic vessel endothelial hyaluronan receptor 1-positive tissue-resident macrophages cooperate with TAMs to establish spatially segregated immunosuppressive niches (111). In glioblastoma (GBM), elevated expression of the hyperlactylation-related gene S100A6 within the hypoxic tumor core suggests intensified local metabolic reprogramming accompanied by enhanced immune-evasion capacity (112).

Under estrogen-deficient conditions, populations of PD-L1+ M2-TAMs, Tregs, and MDSCs are increased in mouse colon cancer models (181). In a melanoma mouse model, gCpG treatment reduces Treg and MDSC abundance (113). HCC accompanied by hepatic fibrosis and abnormal vascular architecture can establish a pathological barrier that promotes compartmentalized accumulation of TAMs and MDSCs, leading to impaired DC maturation and defective antigen cross-presentation (114). These findings indicate that immunosuppressive-cell enrichment is tightly interconnected with metabolic imbalance, impaired antigen presentation, and reduced immune recognition.

### 3.4 Metabolic regulation of immune-checkpoint expression

Metabolic reprogramming can directly regulate the expression of immune-checkpoint molecules, thereby promoting tumor immune evasion at the metabolic level (115). Dysregulated glucose metabolism, lipid metabolic remodeling, and amino acid metabolic remodeling can converge on checkpoint-regulatory pathways, particularly those controlling PD-L1 expression (116). The key feature of this process is the integration of checkpoint expression into the regulatory output of metabolic reprogramming.

Glycolytic reprogramming can function as an upstream driver of checkpoint regulation. Lactylation can inhibit lysosomal degradation of PD-L1 and enhance PD-L1 expression through activation of acetyl-CoA acetyltransferase 2 transcription (117). NCAPH increases glycolytic flux and lactate accumulation while stabilizing PD-L1 protein through inhibition of ubiquitin-mediated degradation (118). Noncoding RNAs can promote glycolysis and lactate secretion, establish an acidic microenvironment, and activate the TEAD/TAZ-signaling pathway, ultimately increasing PD-L1 expression (119). The Kyn–AhR–IDO1 axis participates in remodeling checkpoint-expression programs (120), whereas CD36-mediated lipid metabolic imbalance can further promote PD-L1 expression (116, 121).

Beyond PD-1/PD-L1 and CTLA-4, emerging inhibitory receptors such as LAG-3, TIM-3, TIGIT, VISTA, and B- and T-lymphocyte attenuator are also components of tumor immune-evasion networks (122). ALDH2-mediated aldehyde metabolism directly upregulates VISTA expression on tumor cells through activation of the NOD1/NF-κB-signaling pathway (123). Together, these findings demonstrate that abnormalities in glucose, lipid, and amino acid metabolism can amplify immune-checkpoint expression.

## 4 Integrated feedback circuits linking metabolism and immune evasion

**Figure 2:**
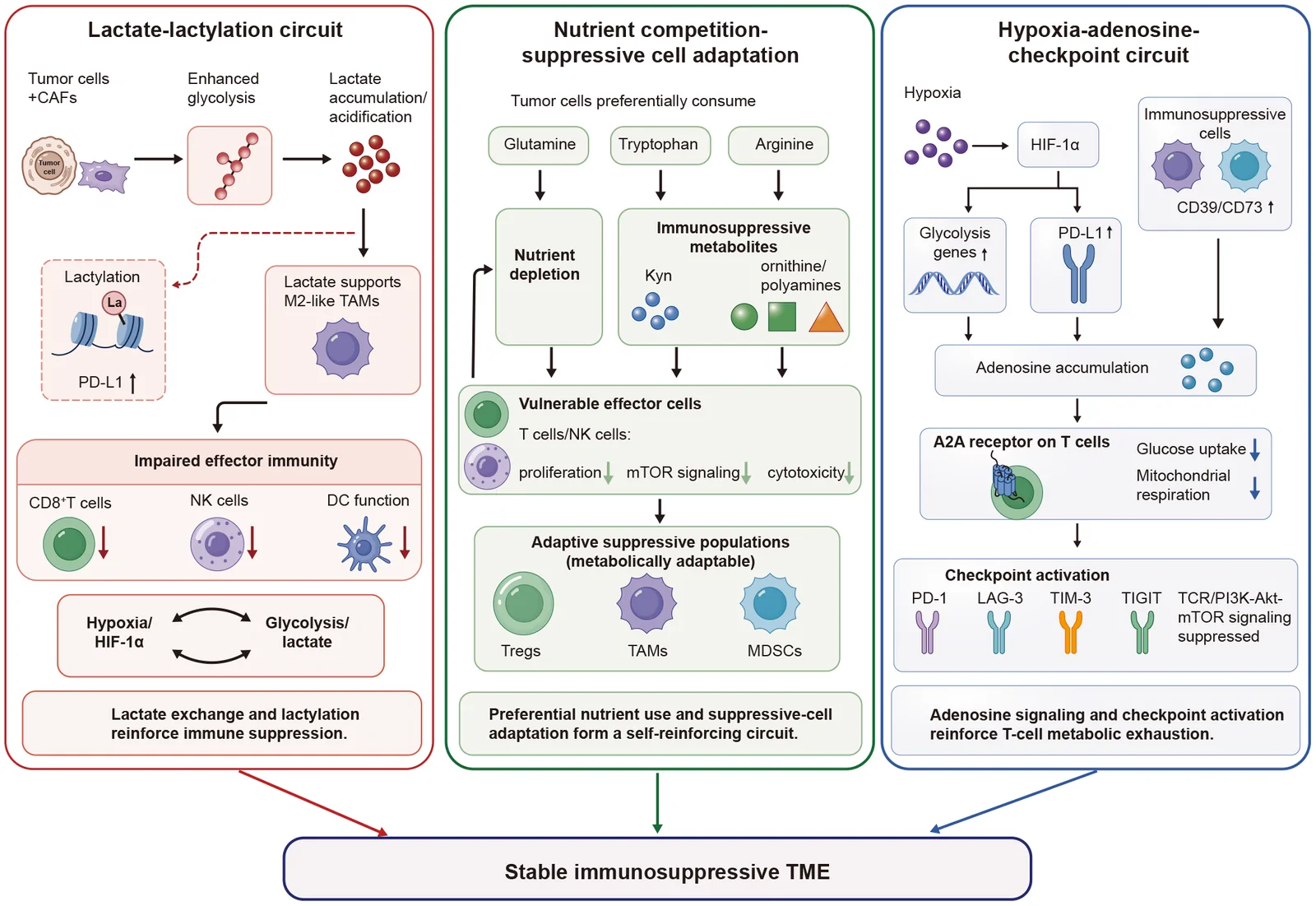
Integrated metabolic circuits linking lactate/lactylation, nutrient competition, and hypoxia–adenosine signaling to immune suppression. The figure illustrates three major metabolic–immune regulatory circuits within the tumor microenvironment. The lactate–lactylation circuit shows how enhanced glycolysis and lactate accumulation promote lactylation-mediated regulation, M2-like tumor-associated macrophage polarization, and impaired effector immune-cell function. The nutrient competition–suppressive cell adaptation circuit demonstrates how preferential consumption of glutamine, tryptophan, and arginine by tumor cells and immunosuppressive populations leads to nutrient depletion, impaired T-cell and natural killer (NK) cell activity, and enrichment of metabolically adaptive regulatory populations, including Tregs, TAMs, and MDSCs. The hypoxia–adenosine–checkpoint circuit depicts how hypoxia induces HIF-1α activation, glycolytic reprogramming, PD-L1 expression, and adenosine accumulation, which activates A2A receptor signaling and promotes T-cell metabolic dysfunction and immune exhaustion. These interconnected pathways collectively contribute to the establishment and maintenance of an immunosuppressive tumor microenvironment. CAFs, cancer-associated fibroblasts; M2-TAMs, M2-like tumor-associated macrophages; Kyn, kynurenine; mTOR, mechanistic target of rapamycin; HIF-1α, hypoxia-inducible factor 1α; PD-L1, programmed death-ligand 1; A2A receptor, adenosine A2A receptor; TIGIT, T-cell immunoreceptor with Ig and ITIM domains; TIM-3, T-cell immunoglobulin and mucin-domain containing-3; LAG-3, lymphocyte activation gene-3.

The metabolic programs described above do not act as isolated suppressive signals. Instead, reciprocal interactions among tumor, cancer-associated fibroblasts (CAFs), and immune cells reinforce lactate accumulation, nutrient depletion, hypoxia, and checkpoint activation, thereby stabilizing an immunosuppressive tumor microenvironment (**Figure 2**).

### 4.1 Lactate–lactylation feedback between tumor, CAFs, and immune cells

CAFs amplify tumor-associated glycolysis through the reverse Warburg effect, increasing lactate accumulation and extracellular acidification. These metabolic changes impair T cell activation, NK cell cytotoxicity, and DC-mediated antigen presentation (93). CAF-derived lactate promotes immunosuppressive M2-like macrophage polarization, whereas CAF-secreted cytokines and chemokines contribute to monocyte recruitment and further reinforce TAM accumulation and polarization (22). In turn, metabolically adapted TAMs reinforce tumor-cell glycolysis and biosynthetic activity, thereby sustaining lactate production and establishing a reciprocal metabolic–immune feedback circuit (124).

Hypoxia-induced HIF-1α activation similarly promotes glycolysis and lactate production in cancer cells, whereas the resulting lactate induces M2-like TAM polarization through G protein-coupled receptor 132 signaling. Polarized M2-like TAMs can, in turn, stabilize HIF-1α in tumor cells through exosomal HISLA lncRNA, further enhancing lactate secretion and forming a bidirectional positive-feedback loop between metabolic remodeling and immune suppression (125).

Lactylation can convert this metabolite-rich environment into sustained transcriptional and phenotypic changes. CAF-derived lactate promotes H3K18 lactylation and enhances PD-L1 expression through the NCAPG/SRC/STAT3-signaling axis, whereas lactate-associated H3K14 and H3K18 lactylation activates Wnt/β-catenin, IκBα/NF-κB, and PD-L1-related pathways, suppressing cytotoxic T-lymphocyte activation and infiltration (34, 108, 126). Thus, lactate exchange and lactylation jointly sustain reciprocal metabolic and immune suppression.

### 4.2 Nutrient competition–immunosuppressive cell adaptation circuit

Tumor cells preferentially consume glutamine, tryptophan, and arginine to support biosynthesis, energy metabolism, and redox homeostasis. Enhanced glutamine utilization, IDO1/TDO2-mediated tryptophan degradation, and ARG1/2-mediated arginine metabolism reduce local nutrient availability and generate immunosuppressive metabolites, thereby impairing T cell proliferation, mTOR signaling, and cytotoxic activity (12, 28, 127).

These nutrient-restricted conditions exert unequal effects on immune-cell populations. Effector T cells and NK cells are vulnerable to metabolic insufficiency, whereas Tregs, TAMs, and MDSCs exhibit greater metabolic flexibility and can maintain suppressive activity through fatty acid oxidation, glycolysis, or amino acid metabolic remodeling (41, 92). This differential adaptation favors the enrichment and persistence of immunosuppressive cells.

The selected immunosuppressive populations further reinforce nutrient depletion. Metabolically adaptable Tregs, TAMs, and MDSCs maintain suppressive activity under nutrient-restricted conditions, whereas ARG1/2-expressing MDSCs and M2-like TAMs promote arginine depletion and produce ornithine, urea, polyamines, and proline (49, 51, 128). Consequently, preferential tumor-cell nutrient use and the metabolic activities of immunosuppressive cells form a self-reinforcing circuit that progressively weakens antitumor immunity.

### 4.3 Hypoxia–adenosine–checkpoint feedback circuit

Hypoxia intensifies glycolysis, lactate accumulation, nutrient limitation, and immune-checkpoint expression. HIF-1α promotes glucose uptake, lactate production, and extracellular acidification through the upregulation of GLUT1/GLUT3, HK2, LDHA, and MCT4 (62, 63, 66). IF-1α-dependent pathways increase PD-L1 expression, while hypoxia-associated signaling can further interact with other immune-checkpoint pathways, including CTLA-4 and LAG-3 programs (78–81).

Under hypoxic conditions, adenosine accumulation activates immunosuppressive signaling through the adenosine A2A receptor, while elevated expression of CD39 and ecto-5′-nucleotidase (CD73) on immunosuppressive cells further promotes adenosine production. Tumor-derived adenosine suppresses T cell activation and metabolic fitness by inhibiting glucose uptake and mitochondrial respiration, thereby promoting T cell dysfunction (129, 130).

Checkpoint activation further aggravates this metabolic dysfunction. LAG-3, TIM-3, and TIGIT suppress proximal T cell receptor signaling, downregulate the PI3K/Akt/mTOR pathway, reduce glucose metabolic flux, and cooperate with PD-1 signaling to worsen mitochondrial dysfunction and metabolic exhaustion (80). Thus, hypoxia-driven adenosine accumulation and checkpoint signaling reciprocally reinforce T cell metabolic suppression and stabilize the immunosuppressive microenvironment.

## 5 Therapeutic targeting of core metabolic–immune circuits

The metabolic–immune circuits described above provide several therapeutic entry points. Accordingly, this section organizes pharmacological and molecular interventions around the lactate–lactylation axis, nutrient competition and amino acid metabolism, and the hypoxia–adenosine–checkpoint axis. Representative agents, model systems, evidence maturity, and trial phase or registration information are summarized in **Table 1**.

**Table 1:** Representative pharmacological and molecular interventions targeting core metabolic–immune circuits.

Metabolic axis	Target/pathway	Agent/intervention	Mechanism of immune modulation	Cancer type/model	Evidence stage	Clinical status (phase/NCT)	Reference(s)
Lactate–lactylation	MCT4-mediated lactate export	MCT4 silencing (siMCT4)	Blocks lactate efflux, reduces extracellular lactate, disrupts metabolic symbiosis, and promotes M1 macrophage polarization.	4T1 breast cancer; cell and mouse models	Preclinical	—	240
Lactate–lactylation	LDHA-mediated lactate production	Ldha knockdown	Reduces glucose consumption and lactate production and decreases MDSC abundance and uPAR/cholesterol-associated suppressive phenotypes.	PDAC mouse model and human tumor samples	Preclinical	—	241
Lactate–lactylation	LDHA–H4K5la–PRC2–MHC-I axis	FX-11 plus anti-PD-1	Reduces H4K5 lactylation and PRC2-mediated MHC-I repression, restoring antigen presentation and sensitizing tumors to PD-1 blockade.	SCLC-A cell and mouse models	Preclinical	—	296
Metabolic epigenetic regulation	FTO-mediated glycolytic regulation	Dac51 plus anti-PD-L1	Inhibits FTO-dependent glycolytic reprogramming, increases intratumoral CD8+ T cells, and enhances PD-L1 blockade.	B16-OVA melanoma, MC38 colorectal, and LLC models	Preclinical	—	244
Epigenetic regulation of immune recognition	Pan-HDAC activity/antigen presentation	Panobinostat (LBH589)	Upregulates MHC-I/II programs, promotes dendritic-cell maturation, and increases CD4+ T cell, CD8+ T cell, and NK-cell proportions.	4T1 TNBC cell and mouse models	Preclinical	—	297
Glutamine metabolism	GLS1	Telaglenastat (CB-839) plus nivolumab	Restricts glutaminolysis and tests whether tumor metabolic inhibition can enhance PD-1 blockade.	Metastatic melanoma, ccRCC, and NSCLC	Clinical	Phase I/II; NCT02771626	298
Glutamine metabolism	GLS1/glutamine utilization	BPTES plus anti-PD-L1	Glutamine restriction increases tumor-cell PD-L1/Fas signaling; combined PD-L1 blockade enhances T cell-mediated killing.	Cancer cell and syngeneic mouse models	Preclinical	—	299
Glutamine metabolism	Broad glutamine metabolism	Sirpiglenastat (DRP-104) plus trametinib	Induces a tumor metabolic crisis; ERK-pathway inhibition counters adaptive signaling and improves tumor control.	PDAC models; advanced solid tumors/NSCLC trials	Preclinical and clinical	Phase I/IIa, NCT04471415; Phase II, NCT07249372	300
Tryptophan–Kyn metabolism	IDO1	Epacadostat plus pembrolizumab	Inhibits IDO1-mediated Kyn production; phase III testing did not improve PFS or OS over pembrolizumab alone.	Unresectable/metastatic melanoma	Clinical	Phase III; NCT02752074	301
Tryptophan–Kyn metabolism	IDO pathway	Indoximod-based chemo-immunotherapy	Modulates IDO-pathway-associated immune suppression in combination with chemotherapy and radiotherapy.	Pediatric recurrent brain tumors and newly diagnosed DIPG	Clinical	Phase I; NCT02502708	302
Tryptophan–Kyn metabolism	IDO1	BGB-5777 plus anti-PD-1 and radiotherapy	Combines IDO1 inhibition, checkpoint blockade, and radiation to increase tumor-infiltrating T cells and prolong survival.	Advanced orthotopic GBM mouse models	Preclinical	—	303
Tryptophan–Kyn metabolism	IDO1	Navoximod (NLG919/GDC-0919) plus atezolizumab	Reduces IDO1 activity and Kyn-mediated suppression in combination with PD-L1 blockade.	Advanced solid tumors	Clinical	Phase I; NCT02471846	304
Tryptophan–Kyn metabolism	Kynurenine degradation	Engineered PEGylated KYNase variants	Directly hydrolyzes immunosuppressive Kyn and reduces Kyn exposure within the tumor microenvironment.	Cancer cell and mouse models	Preclinical	—	305
Arginine metabolism	Arginine-responsive TEAD4/OXPHOS program	TEAD4 targeting plus arginine deprivation	Disrupts arginine-dependent OXPHOS transcription and enhances sensitivity to arginine deprivation.	Prostate cancer cell and xenograft models	Preclinical	—	250
Polyamine–adenosine crosstalk	Polyamine synthesis and NT5E/CD73	DFMO plus NT5E/CD73 knockdown	Reduces polyamine metabolism and adenosine-associated CAF immunosuppression, producing greater tumor suppression than either intervention alone.	PDAC cell and xenograft models	Preclinical	—	246
Hypoxia–adenosine–checkpoint	CD73–adenosine pathway	Oleclumab plus durvalumab	Blocks CD73-dependent adenosine generation while inhibiting PD-L1; updated phase II data showed a PFS signal without an established OS association.	Unresectable stage III NSCLC after cCRT	Clinical	Phase II; NCT03822351	306

Evidence stage, trial phase, and registration information reflect the cited original studies and ClinicalTrials.gov records available at the time of revision. “—” indicates that no clinical phase or registration number applies to a preclinical intervention. B16-OVA, ovalbumin-expressing B16 melanoma; CAF, cancer-associated fibroblast; cCRT, concurrent chemoradiotherapy; ccRCC, clear-cell renal cell carcinoma; CD73, ecto-5′-nucleotidase; DFMO, difluoromethylornithine; DIPG, diffuse intrinsic pontine glioma; ERK, extracellular signal-regulated kinase; FTO, fat mass and obesity-associated protein; GBM, glioblastoma; GLS1, glutaminase 1; IDO1, indoleamine 2,3-dioxygenase 1; Kyn, kynurenine; KYNase, kynureninase; LDHA, lactate dehydrogenase A; LLC, Lewis lung carcinoma; MCT4, monocarboxylate transporter 4; MDSC, myeloid-derived suppressor cell; MHC-I, major histocompatibility complex class I; MHC-II, major histocompatibility complex class II; NSCLC, non-small-cell lung cancer; NT5E, ecto-5′-nucleotidase gene; OS, overall survival; OXPHOS, oxidative phosphorylation; PD-1, programmed cell death protein 1; PD-L1, programmed death-ligand 1; PDAC, pancreatic ductal adenocarcinoma; PFS, progression-free survival; PRC2, polycomb repressive complex 2; SCLC-A, ASCL1-high small-cell lung cancer; TEAD4, TEA domain transcription factor 4; TNBC, triple-negative breast cancer; uPAR, urokinase plasminogen activator receptor.

### 5.1 Targeting the lactate–lactylation axis

Accumulating preclinical evidence suggests that inhibition of lactate production or transport can partially restore antitumor immunity. In a 4T1 subcutaneous breast cancer model, MCT4 silencing blocked lactate efflux, disrupted tumor metabolic symbiosis, promoted M1 macrophage polarization, and enhanced antitumor immune responses (131). In pancreatic ductal adenocarcinoma (PDAC), LDHA knockdown reduced glucose consumption and lactate production, decreased MDSC abundance, and attenuated cholesterol-metabolism- and urokinase plasminogen activator receptor (uPAR)-associated phenotypes in monocytic MDSCs; concordant associations were observed in human PDAC samples (132).

More recent work has linked LDHA inhibition directly to immune-checkpoint sensitization. In ASCL1-high small-cell lung cancer (SCLC-A) models, genetic or pharmacological LDHA inhibition restored MHC-I antigen presentation by reducing H4K5 lactylation and polycomb repressive complex 2 (PRC2)-associated repression; FX-11 combined with anti-PD-1 therapy synergistically suppressed tumor growth and increased immune activation *in vivo* (133). In bladder cancer, LDHA-driven lactate production promoted EP300-mediated H4K5 lactylation and PD-L1 transcription, and LDHA overexpression reduced CD8^+^ T cell cytotoxicity and sensitivity to PD-1 blockade (40). These findings support lactate production as a therapeutic target, although current evidence remains preclinical.

Strategies that increase lactate decomposition or neutralize the acidic microenvironment may alleviate lactate-associated immune suppression. The bimetallic nanozyme PEG@AuCZ@CC enhances DC maturation and promotes CD4^+^/CD8^+^ T cell effector functions by increasing lactate decomposition (134). CaO_2_ nanoparticles suppressed tumor growth by neutralizing the acidic microenvironment while simultaneously alleviating hypoxia and lactate accumulation (135).

Beyond directly targeting lactate production or transport, modulation of metabolism-associated epigenetic regulators may provide an additional strategy to reverse immune suppression. The N6-methyladenosine (m6A) demethylase fat mass and obesity-associated protein (FTO) promotes tumor glycolysis and restricts CD8^+^ T cell activation; Dac51 inhibited FTO, increased intratumoral CD8^+^ T cell infiltration, and synergized with anti-PD-L1 treatment in B16-OVA and MC38 models (136). Panobinostat (LBH589), a pan-HDAC inhibitor rather than a lactylation-specific agent, upregulated MHC-I/II antigen-presentation programs, promoted dendritic-cell maturation, and increased CD4^+^ T cell, CD8^+^ T cell, and NK cell proportions in a 4T1 triple-negative breast cancer (TNBC) model (137).

Although lactate-directed intervention is supported by a relatively clear mechanistic rationale, current evidence remains largely derived from *in vitro* and preclinical animal studies. Its translational value therefore depends on appropriate patient stratification, effective toxicity management, rational combination with immunotherapeutic approaches, and consideration of tumor metabolic heterogeneity.

### 5.2 Targeting nutrient competition

#### 5.2.1 Glutamine metabolism

GLS inhibition has shown both preclinical immunometabolic effects and limited clinical translation. In preclinical tumor models, inhibition of glutamine utilization with BPTES enhanced T cell-mediated antitumor activity when combined with PD-L1 blockade (138). Telaglenastat (CB-839) has also been evaluated with nivolumab in a phase I/II study involving metastatic melanoma, clear-cell renal cell carcinoma, and NSCLC (NCT02771626). The combination was generally tolerable, but no sufficiently strong efficacy signal emerged across cohorts (139). By contrast, the broad glutamine antagonist sirpiglenastat (DRP-104) produced a metabolic crisis in PDAC models, with compensatory ERK activation; combination with trametinib improved tumor control and survival (140). DRP-104 has entered phase I/IIa evaluation in advanced solid tumors (NCT04471415) and phase II evaluation in NFE2L2/KEAP1-altered NSCLC (NCT07249372). Glutamine-directed intervention must nevertheless account for immune-cell dependence on this nutrient: SLC38A2-high tumor cells can deplete glutamine and impair cDC1 survival and function (141). Thus, preserving immune-cell metabolic fitness may be as important as suppressing tumor glutamine use.

#### 5.2.2 Tryptophan–IDO1/TDO2–kynurenine axis

Clinical experience with the tryptophan–IDO1 axis has been heterogeneous. Epacadostat combined with pembrolizumab failed to improve progression-free or overall survival over pembrolizumab alone in the phase III ECHO-301/KEYNOTE-252 melanoma trial (NCT02752074) (301), although recent preclinical data in microsatellite-stable CRC suggest that IDO1-targeted strategies may retain context-dependent activity (142). Indoximod-based chemo-immunotherapy was feasible in a first-in-children phase I study of pediatric brain tumors (NCT02502708), providing early clinical evidence for an IDO-pathway modulator outside melanoma (143). Navoximod (NLG919/GDC-0919) plus atezolizumab was tolerable in a phase I study of advanced solid tumors (NCT02471846), but its activity was not clearly greater than expected with PD-L1 blockade alone (144).

Preclinical strategies continue to explore more complete disruption of Kyn-mediated immune suppression. In advanced GBM models, BGB-5777 combined with radiotherapy and anti-PD-1 therapy increased tumor-infiltrating T cells and prolonged survival (145). Enzymatic depletion of Kyn represents an alternative to blocking its production: a protein-language-model-guided study identified highly active kynureninases and showed that PEGylated KYNase variants reduced Kyn and produced antitumor activity *in vivo* (146). These findings indicate that response depends on tumor subtype, pathway redundancy, pharmacodynamic target engagement, and the immune landscape.

#### 5.2.3 Arginine and myeloid metabolic pathways

Targeting TEA domain transcription factor 4 enhanced the efficacy of arginine-deprivation therapy in prostate cancer cells and xenografts by disrupting an arginine-responsive OXPHOS program, supporting a potential metabolic combination strategy (147). In PDAC models, NT5E/CD73 knockdown reduced adenosine-associated immunosuppression, and combination with the polyamine-synthesis inhibitor difluoromethylornithine produced greater tumor suppression than either intervention alone (148). Both approaches remain preclinical.

#### 5.2.4 Translational challenges of nutrient-targeted therapy

Therapeutic intervention in amino acid metabolism does not follow the principle that stronger metabolic inhibition necessarily results in better outcomes. Tumor and immune cells can share dependence on the same substrates, and the biological effects of metabolic intervention may vary according to tumor genotype and immune-cell vulnerability. For example, increased GSH levels promote liver metastasis in TP53-mutant tumors, whereas the opposite effect has been observed in TP53 wild-type tumors (149). Thus, nutrient-targeted strategies require clearly defined tumor metabolic dependencies and immune-cell vulnerabilities.

### 5.3 Targeting the hypoxia–adenosine–checkpoint axis

Hypoxia is an upstream feature that reinforces lactate accumulation, adenosine production, and immune-checkpoint signaling. Although hypoxia-activated prodrugs and hypoxia-responsive delivery systems remain under investigation, the most clinically advanced intervention within this axis is blockade of adenosine production or signaling, particularly through CD73. This approach is intended to reduce extracellular adenosine and relieve metabolic suppression of effector lymphocytes.

Preclinical PDAC data show that inhibition of CD73 encoded by NT5E blocks adenosine generation and alleviates CAF-associated immunosuppression; combination with difluoromethylornithine further enhances tumor suppression (148). Clinically, the anti-CD73 antibody oleclumab combined with durvalumab was evaluated in the randomized phase II COAST trial in unresectable stage III NSCLC after concurrent chemoradiotherapy (NCT03822351). Updated results showed longer progression-free survival with the combination than with durvalumab alone, whereas an overall-survival association was not established (150).

Together, these data support combination strategies that pair metabolic intervention with checkpoint blockade, including FX-11 plus anti-PD-1 (133), glutamine-targeted therapy plus PD-1/PD-L1 blockade (138, 139), IDO1 or Kyn-degrading approaches plus checkpoint inhibition (143–146, 151), and CD73 blockade plus durvalumab (150). However, efficacy remains variable and context dependent, and clinically meaningful target engagement, patient selection, and toxicity management remain major challenges.

### 5.4 Emerging strategies supporting metabolic–immune therapy

#### 5.4.1 Metabolically engineered cell therapies

Metabolic reprogramming of chimeric antigen receptor T (CAR-T) cells can enhance glutamine uptake, improve metabolic fitness, and strengthen cytotoxic activity, thereby partially overcoming the suppressive effects of the tumor microenvironment (152). Although CAR-T therapy has demonstrated substantial clinical efficacy in hematologic malignancies (131), its application in solid tumors remains limited, primarily because of insufficient tumor infiltration, poor cellular persistence, and progressive functional exhaustion (153). Immunosuppressive components enriched within the solid tumor TME, including CAFs, Tregs, and TAMs, further impair CAR-T cell activation and long-term therapeutic responses (154).

Localized immunomodulation and mitochondrial remodeling have been explored to improve engineered-cell function. A bicistronic vector coexpressing CAR and a PD-1-blocking scFv enables CAR-T cells to secrete functional scFv locally, enhancing IFN-γ secretion and reducing PD-L1-mediated suppression of proliferation (155). CAR-T cells coexpressing a modified form of peroxisome proliferator-activated receptor γ coactivator-1α exhibit enhanced mitochondrial biogenesis and improved effector function in solid tumor models (105).

#### 5.4.2 Targeted delivery and nanotechnology

Nanocarrier systems can improve drug solubility and stability, enable targeted delivery, and support coloading of metabolic modulators and immunoregulatory agents, thereby increasing local drug exposure while reducing off-target effects and systemic toxicity (47, 134, 156). In pancreatic cancer models, a glutamine nutrition-functionalized iron delivery system loaded with the glutamine transporter inhibitor V9302 enhanced cellular endocytosis, induced ferroptosis, and achieved controlled drug release (157). Depot-type nanoparticles delivering prekynureninase reduced Kyn levels in TME, restored effector T cell activity, and synergized with PD-1 blockade (158). Smart nanosystems co-delivering doxorubicin and NLG919 simultaneously induced immunogenic cell death and regulated amino acid metabolism to alleviate local immunosuppression (159).

Additional platforms directly address the lactate-rich and acidic microenvironment. RGD-Lipo@GOx demonstrated specific antitumor activity in TNBC models through a histone lactylation-associated pathway (160), whereas CaO_2_ nanoparticles neutralized the acidic microenvironment and alleviated hypoxia and lactate accumulation (135). Clinical translation remains constrained by formulation stability, batch-to-batch consistency, controllable *in vivo* biodistribution, and long-term biosafety (161).

#### 5.4.3 Other emerging platforms

Cancer vaccines may be more suitable as components of combination strategies when integrated with approaches that improve antigen presentation or alleviate metabolic immunosuppression (14, 103, 162). Gene-editing technologies are currently used mainly for mechanistic dissection and model validation of metabolism–immunity interactions, while clinical application remains limited by delivery efficiency, off-target effects, long-term safety, and ethical regulation (3, 28).

Engineered bacteria exploit selective colonization of hypoxic tumor tissues to deliver therapeutic payloads or remodel the local immune microenvironment (163, 164). In PDAC models, engineered bacteria combined with ICIs can disrupt tumor-cell ferroptosis defense mechanisms and promote M1-TAM infiltration (163). Oncolytic herpes simplex viruses expressing a humanized PD-1 scFv can increase effector T cell infiltration and suppress Treg expansion, although these approaches primarily enhance local immunogenicity rather than directly target metabolic pathways (165).

### 5.5 Evidence maturity and barriers to clinical translation

Clinical translation targeting the metabolic–immune axis has evolved from single-drug inhibition toward platform-based delivery systems, rational combination therapies, and stratified therapeutic evaluation. Current clinical trials focus on key metabolic enzymes, including PKM2, LDH, FASN, HMGCR, GLS1, and IDO1, as well as glycolysis, glutamine metabolism, and cholesterol metabolism. Small-molecule inhibitors such as 3-bromopyruvate, and CB-839 have entered different stages of clinical evaluation (166). Nevertheless, most approaches remain exploratory, and therapeutic efficacy is inconsistent across tumor types and patient populations.

The lack of mature biomarker systems remains a major barrier to monitoring, patient stratification, and therapeutic prediction. In patients with HCC after trans arterial chemoembolization, serum lactate dehydrogenase levels positively correlate with soluble PD-L1 levels, suggesting a potential association between lactate metabolism and immune status (167). In TNBC, GLUT1, LDHA, PD-L1, and colony-stimulating factor 1 have similarly been associated with immune-exclusion phenotypes (168). However, these associations remain at the candidate-biomarker level and have not established stable, minimally invasive, and dynamically monitorable clinical evaluation systems.

At the current stage, the main challenge is not the lack of candidate targets but how to integrate metabolic heterogeneity, immune status, delivery efficiency, and treatment-associated toxicity into a clinically actionable framework. Rational combination strategies, dynamic monitoring, and stratified evaluation are therefore central to further clinical development.

## 6 Discussion

Tumor metabolic reprogramming and immune evasion should be considered as interconnected processes rather than independent biological events. In this review, lactate/lactylation and nutrient competition represent two complementary metabolic–immune regulatory axes. Lactate accumulation reflects not only altered enhanced glycolytic metabolism but also a signaling and epigenetic mechanism through which metabolic changes can influence immune-cell function and tumor immune escape (5–8, 23, 24, 35–37). In parallel, nutrient competition represents a resource-limiting mechanism in which tumor cells reshape the availability of amino acids and lipids, thereby affecting the metabolic fitness of effector immune cells and favoring the maintenance of immunosuppressive populations (4, 9, 10, 41). Together, these two axes provide a framework for understanding how metabolic alterations are translated into immune dysfunction within the TME.

The interaction between lactate/lactylation and nutrient competition is not simply a parallel relationship but reflects different layers of metabolic regulation. Increased glycolytic activity promotes glucose consumption and lactate production, thereby simultaneously altering nutrient availability and generating metabolite-mediated signaling changes within the TME. Lactate-enriched conditions can impair CD8^+^ T cell function through metabolic reprogramming, disruption of T cell receptor signaling, and interference with intracellular metabolic homeostasis (25–27). In addition, lactate accumulation and extracellular acidification contribute to immunosuppressive remodeling of the TME by promoting M2-like macrophage polarization, reducing NK cell activity, and suppressing T cell activation (28). Meanwhile, lactate-derived lysine lactylation provides a mechanism by which metabolic alterations are translated into transcriptional programs associated with immune regulation, including regulation of PD-L1 expression and macrophage polarization (7, 8, 35–40). Therefore, nutrient competition primarily reflects changes in substrate availability, whereas lactate-associated signaling and lactylation represent additional regulatory mechanisms through which metabolic alterations shape immune-cell states within the TME.

Current controversies in tumor immunometabolism mainly arise from the context-dependent effects of metabolic alterations, challenging the traditional view that tumor-associated metabolites uniformly promote immune suppression. Despite substantial evidence supporting the immunosuppressive effects of tumor-derived lactate, recent studies indicate that lactate does not function as a uniformly inhibitory metabolite. Under specific experimental conditions, lactate can promote memory-like and stem-like characteristics of CD8^+^ T cells and enhance antitumor immunity, suggesting that lactate may exert distinct effects depending on cellular metabolic state and immune-cell context (169). Similar context dependency has also been observed for other metabolic regulators. HIF-1α generally promotes tumor adaptation and immune suppression by enhancing glycolysis, lactate production, and immune-checkpoint expression; however, it can also support immune-cell survival and functional maintenance in specific physiological environments, such as promoting the survival of lung tissue-resident T cells (84–86). Succinate may facilitate dendritic-cell maturation and activation at appropriate concentrations, whereas excessive accumulation can promote tolerogenic responses through HIF-1α-associated pathways (106, 107). Moreover, glutathione-related metabolic effects may vary according to tumor genetic background, as increased GSH levels promote liver metastasis in TP53-mutant tumors but exhibit different effects in TP53-wild-type tumors (149). Collectively, these findings indicate that metabolic alterations should not be interpreted as universally immunosuppressive mechanisms but rather as context-dependent regulators. Their biological effects are shaped by the interaction among metabolite concentration, cellular identity, genetic background, and tissue environment. Therefore, understanding the specific conditions under which metabolic pathways promote or restrain antitumor immunity is essential for developing effective metabolism-targeted therapeutic strategies.

Although metabolic–immune interventions have shown promising therapeutic potential, their clinical translation remains challenging. The major limitation is not the absence of candidate targets but the difficulty in distinguishing tumor-specific metabolic dependencies from pathways required for immune-cell function. For example, glutamine metabolism supports tumor growth but is also important for immune-cell survival and antigen-presenting cell function, indicating that broad metabolic inhibition may produce unpredictable effects (141, 170, 171). In addition, metabolic plasticity enables tumor cells and immune cells to adapt through dynamic metabolic reprogramming, creating additional challenges for therapeutic targeting (172). Consequently, future clinical strategies will require biomarker-guided patient selection, integration of metabolic and immune profiling, and rational combination approaches rather than nonspecific metabolic blockade.

## 7 Conclusion

Taken together, lactate/lactylation and nutrient competition constitute two complementary mechanisms through which tumor metabolic reprogramming is translated into immune evasion. Glycolysis-derived lactate integrates metabolic coupling, extracellular acidification, signaling, and epigenetic regulation, whereas depletion of glutamine, tryptophan, and arginine and altered lipid availability reduce the metabolic fitness of effector cells and favor Tregs, TAMs, and MDSCs. Hypoxia further amplifies these processes and links them to adenosine accumulation and immune-checkpoint activation, generating self-reinforcing circuits that impair cytotoxicity, antigen presentation, and immune recognition within the TME.

These pathways should not be interpreted as uniformly immunosuppressive. Their effects vary with metabolite concentration, cellular identity, tumor genotype, and tissue context, which may partly explain the heterogeneous responses to metabolism-directed therapies. Although targeting lactate production or transport, lactylation-associated regulators, amino acid metabolism, and the CD73–adenosine axis has shown mechanistic promise, most supporting evidence remains preclinical, and clinical results have been variable. Broad metabolic inhibition may also compromise the metabolic requirements of antitumor immune cells.

Future studies should therefore prioritize clinically actionable metabolic dependencies rather than expanding the list of candidate targets. Biomarker-guided patient selection, confirmation of intratumoral target engagement, preservation of immune-cell metabolic fitness, and rational combination with immune checkpoint inhibitors will be essential. Integration of single-cell and spatial analyses, isotope tracing, and longitudinal clinical sampling may define when and where lactate/lactylation or nutrient competition becomes a dominant driver of immune escape and help identify patients most likely to benefit from metabolic–immune interventions.
